# A comparative study of suction blister epidermal grafting and automated blister epidermal micrograft in stable vitiligo

**DOI:** 10.1038/s41598-021-04299-0

**Published:** 2022-01-10

**Authors:** Pei-Rong Gao, Chi-Hui Wang, Yu-Jr Lin, Yu-Huei Huang, Ya-Ching Chang, Wen-Hung Chung, Chau Yee Ng

**Affiliations:** 1 Department of Dermatology, Chang Gung Memorial Hospital, Linkou Branch, No.5, Fushin Street, Taoyuan, Keelung Branch, Linkou, Taipei Taiwan; 2 College of Medicine, Chang Gung University, Taoyuan, Taiwan; 3 Department of Biomedical Engineering, Chung Yuan Christian University, Chung-Li, Taiwan; 4 Clinical Informatics and Medical Statistics Research Center, Chang Gung University, Taoyuan City, Taiwan; 5 Graduate Institute of Clinical Medical Sciences, Chang Gung University, Taoyuan, Taiwan; 6 Vitiligo Clinic and Research Center, Chang Gung Memorial Hospital, Linkou, Taiwan

**Keywords:** Skin diseases, Quality of life, Therapeutics, Outcomes research, Clinical trial design

## Abstract

The automated blister epidermal micrograft (ABEM) is a newly introduced surgical transplantation for refractory vitiligo. Comparative analysis of other surgical methods is lacking. We conducted a retrospective study to compare the efficacy, safety, and experience of ABEM with conventional suction blister epidermal graft (SBEG). A total of 118 anatomically based vitiligo lesions from 75 patients were included. The primary outcome was the degree of repigmentation; the patient and operator experience were evaluated. SBEG had a significantly greater incidence of repigmentation (p < 0.001), as measured by the Physician Global Assessment, as well as improvements in the Vitiligo Area Scoring Index, particularly on the face/neck area (p < 0.001). ABEM, on the contrary, had reduced donor harvest time, a better patient operative experience, and more significant Dermatology Life Quality Index improvements. In a subgroup of 38 lesions from ten patients who received both SBEG and ABEM concomitantly, there was no difference in the degree of repigmentation in the same recipient area. Overall, the degree of repigmentation for SBEG is higher than ABEM, especially in the mobilized region, and the cost is less expensive. On the contrary, ABEM requires less procedure learning curve and can supply a greater transplanting zone with shorter donor site recovery. Understanding the benefits and drawbacks of two blister grafting procedures is essential for optimal surgical outcomes for vitiligo grafting.

## Introduction

Vitiligo is an acquired depigmentation of the skin caused by the complete loss of melanocytes. It affects approximately 0.5 to 2 percent of the general population^1^. The characteristic clinical features are white macules and patches with sharply circumscribed but irregular borders. Various modalities, both conventional and alternative, non-surgical and surgical, are used in the treatment. Vitiligo has a detrimental effect on a patient's quality of life due to social stigma and comorbidities^2–6^.

Vitiligo is one of the most complex dermatological conditions to manage. The recommended first-line treatment is topical steroids, either alone or in combination with topical calcineurin inhibitors^1,7^. Systemic steroids or immunosuppressants are used to halt the autoimmune destruction for rapidly progressive unstable lesions. Narrowband ultraviolet B phototherapy and monochromatic excimer laser can enhance melanocyte regeneration and immunomodulatory effect^1,7^. Patients suffering from stable recalcitrant lesions that have not responded to non-surgical methods may opt for surgical treatment using melanocyte transplant techniques^1,7^. The two most common types of procedures are tissue grafts and cellular grafts. Melanocyte-rich tissue grafting includes full-thickness, split-thickness, and suction blister grafts^7–9^. A novel automated epidermal harvesting technique that induces blister epidermal micrograft formation is now commercially available^10^.

This study aims to compare conventional suction blister epidermal graft (SBEG) with automated blister epidermal micrograft (ABEM) technique for patients with stable vitiligo in terms of efficacy, safety, and patient and operator experience. In addition, anatomical areas, including the face, trunk, limbs, and acral parts, were also analyzed. To our knowledge, this is the first study that compares two blister epidermal graft surgical methods for the treatment of stable vitiligo.

## Methods

### Study design

We conducted a retrospective comparative trial from January 2017 to December 2020 in the Dermatology Department of Chang Gung Memorial Hospital, Linkou, Taoyuan, Taiwan. The protocol was approved by the Chang Gung Medical Foundation Institutional Review Board (No.: 202100274B0) before the initiation of the study. All of the research was carried out in accordance with the applicable rules and regulations.

### Patient selection

Patients were recruited from the practice of a single dermatologist in a tertiary medical center. The study included patients with stable vitiligo (both segmental and nonsegmental) who failed to respond to other therapies in the past two years. Stable vitiligo was defined as no deterioration of old lesions and no new lesions within a year. Exclusion criteria included keloidal tendency, active infections, pregnancy, lactation, bleeding diathesis, or any sign of unstable disease (inflammatory or poorly defined border, confetti-like or pentachrome lesion, and Koebner phenomenon). The efficacy and safety of ABEM in segmental and non-segmental vitiligo from the same patients in this study has been investigated and reported in the literature^11^.

### Suction blister epidermal graft (SBEG)

Suction blister epidermal grafting is a low-cost, high-effective technique that results in complete repigmentation in 68–70% of cases^12,13^. This method requires creating a subepidermal bulla from the donor site by prolonged vacuum application; then, the roof is surgically removed and transplanted to the recipient site. A suction syringe without a plunger is applied to generate constant negative pressure on the donor site through a 3-way connector. An infrared lamp as a heating source was used to shorten the blistering time. The recipient site was prepared by ablation with Erbium: Yttrium–aluminium-garnet (Er: YAG) laser (ProFractional™, Sciton Inc., California, USA) with a 2-mm spot-sized handpiece at the average fluence of 6.3 J/cm^2^ for optimal graft adherence and uptake. The graft was then transferred after being modified to the proper size and shape and wrapped with a hydrocolloid dressing.

### Automated blister epidermal micrograft (ABEM)

**Figure 1:**
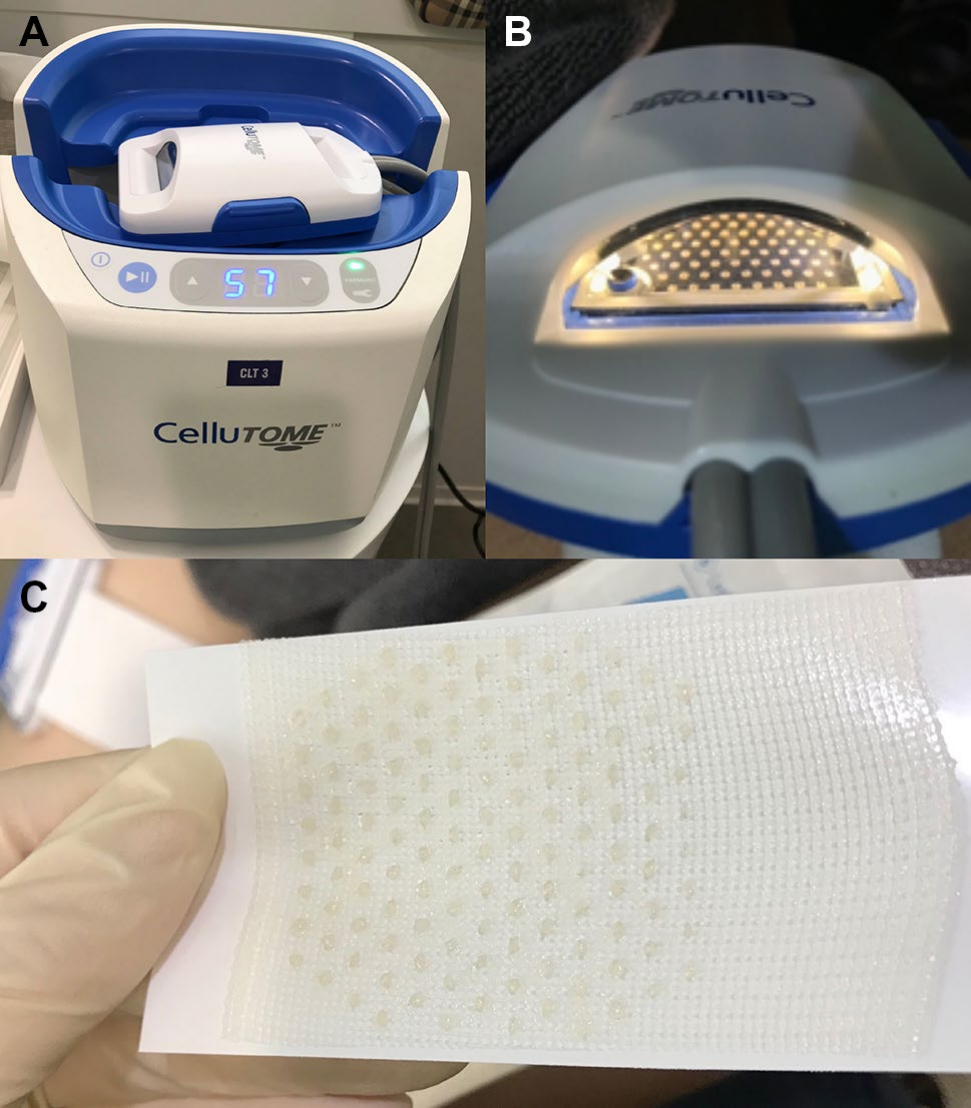
The control unit, vacuum head (**A**), and harvester (**B**) are the three essential components of the automated blister epidermal micrograft system. The vacuum head is subsequently removed, and non-adherent dressing is applied to the grafts (**C**).

The automated blister epidermal micrograft (CelluTome™; Kinetic Concepts, Inc., ACELITY Company, San Antonio, Texas) is a novel option, allowing patients to receive pain-free epidermal skin grafts with reduced donor site trauma (Fig. 1)^14,15^. With a negative pressure of − 400 to − 500 mmHg and a temperature of 37 to 41 °C, the device automatically produces suction micro domes and harvests epidermal micrografts with an area of 20–25 cm^2^^14,15^. A silicone-coated nonadherent dressing (Adaptive Touch™, Systagenix, ACELITY Company, Gargrave, UK) was used to transfer the epidermal micrografts perforated design helps fluid to drain from the micro blisters^16^. After preparing the recipient site with Er: YAG laser ablation, the graft was transferred to the recipient site and wrapped with a hydrocolloid dressing. All patients with ABEM and SBEG received topical tacrolimus 0.1% ointment after grafting procedure. 64% (36/56 anatomical based lesion) with ABEM grafting and 55% (34/62 anatomical based lesion) received excimer light and/or narrow band UVB phototherapy at least once per week after grafting.

### Outcome measures

The primary outcome measure was the degree of repigmentation per lesion. The rate of repigmentation, identified as the area of pigments at the recipient site one year after initial treatment, was graded as very good (≥ 75%), good (50–74%), fair (25–49%), and poor (0–24%) using a Physician Global Assessment (PGA) scale. In addition, improvement in Vitiligo Area Scoring Index (VASI), a quantitative parametric score developed by Hamzavi et al., was used to assess the efficacy^17^. The secondary outcome measure was the patient and operator experience evaluated by operative conditions, pain score, and change in the Dermatology Life Quality Index (DLQI)^18^. Adverse event was monitored and recorded.

### Statistical analysis

Data were analyzed using the R software (R version 3.6.2, 2019 copyright: The R Foundation for Statistical Computing). The descriptive statistic was showed by mean, standard deviation (SD) for continuous variable and count, % for categorical variable.

To compare the results, we used paired sample t-test, chi-square × 2 tests, or Fisher exact tests to compare the difference between various groups. A p-value less than 0.05 was considered statistical significance.

### Ethics approval statement

Reviewed and approved by Chang Gung Medical Foundation IRB; approval #202100274B0.

### Patient consent of publication statement

We obtained written informed consent from all participants.

## Results

In the study, 118 anatomically based lesions from 75 patients with stable vitiligo were enrolled. 62 lesions received conventional suction blister epidermal graft (SBEG), and 56 lesions receiving automated blister epidermal micrograft (ABEM) (Table 1). Their mean age was 34.17 years (range 7–70). The vitiligo lasted an average of 5.89 ± 7.79 years while remaining stable for an average of 4.14 ± 7.12. Patients are all Asians with Fitzpatrick skin types III to IV. The treatment area of ABEM was more extensive than that of SBEG (ABEM 20.09 ± 0.67 cm; SBEG 8.94 ± 5.70, p < 0.001). There were no significant variations between the two groups regarding gender, age, presentation patterns, underlying autoimmune disease, disease duration, disease stability, initial Dermatology Life Quality Index (DLQI), and the initial Vitiligo Area Scoring Index (VASI). The face and neck (74%) were the most commonly grafted area, followed by the trunk (9%) and non-acral limbs (9%).

**Table 1:** Patient characteristics of conventional suction blister epidermal graft (SBEG) with automated blister epidermal micrograft (ABEM).

	Total	SBEG	ABEM	p-value
No. of anatomical based lesions	118	62	56	
**Gender, n (%)**				0.615
F	72 (61%)	36 (58%)	36 (64%)	
M	46 (39%)	26 (42%)	20 (36%)	
Age, years	34.17 ± 14.97	36.69 ± 15.82	31.38 ± 13.56	0.054
**Presentation patterns**				0.781
Segmental	46 (39%)	23 (37%)	23 (41%)	
Non-segmental	72 (61%)	39 (63%)	33 (59%)	
**Underlying disease, n (%)**				0.301
No	106 (90%)	57 (92%)	49 (88%)	
Yes	12 (10%)	5 (8%)	7 (13%)	
Duration of disease, years	5.89 ± 7.79	6.30 ± 9.34	5.45 ± 5.67	0.556
Disease stability, years	4.14 ± 7.12	4.42 ± 8.56	3.83 ± 5.15	0.654
DLQI	5.80 ± 2.88	5.69 ± 3.20	5.91 ± 2.52	0.681
Initial VASI	96.48 ± 9.16	96.69 ± 9.71	96.25 ± 8.59	0.793
Treatment area (cm^2^)	14.23 ± 6.96	8.94 ± 5.70	20.09 ± 0.67	< 0.001
**Treatment localization, n (%)**				0.993
Face/Neck	87 (74%)	45 (73%)	42 (75%)	
Trunk	11 (9%)	6 (10%)	5 (9%)	
Limbs	11 (9%)	6 (10%)	5 (9%)	
Acral	9 (8%)	5 (8%)	4 (7%)	

Underlying disease: Autoimmune disorders including lupus erythematosus, autoimmune thyroiditis, rheumatoid arthritis.

*DLQI* Dermatology life quality index, *VASI* Vitiligo area scoring index.

For the degree of repigmentation after grafting according to the PGA scale, 47 (76%) of the lesions in the SBEG group were scored as very good (≥ 75%); 5 (8%) as good (50–74%); 1 (2%) as fair (25–49%); and 9 (15%) as poor (0–24%) (Table 2). In the ABEM group, 22 (39%) of the lesions were scored as very good; 12 (21%) as good; 13 (23%) as fair; and 9 (16%) as poor. The degree of repigmentation in the ABEM group is significantly lower than in the SBEG group (p < 0.001). When comparing the VASI from baseline, the SBEG group showed significantly more improvement than the ABEM group (p < 0.001). According to the subgroup analysis based on body parts, the changes in VASI were more profound in the SBEG group on the face/neck region compared to ABEM. At the same time, no difference was observed on the trunk, non-acral limbs, or acral area.

**Table 2:** Comparative analysis of the efficacy of conventional suction blister epidermal graft(SBEG) with automated blister epidermal micrograft (ABEM).

	Total	SBEG	ABEM	p-value
No. of anatomical based lesions	118	62	56	
**Repigmentation PGA, n (%)**				< 0.001
Very good (≥ 75%)	69 (58%)	47 (76%)	22 (39%)	
Good (50–74%)	17 (14%)	5 (8%)	12 (21%)	
Fair (25–49%)	14 (12%)	1 (2%)	13 (23%)	
Poor (0–24%)	18 (15%)	9 (15%)	9 (16%)	
% of VASI change	62.05 ± 33.19	74.67 ± 32.63	48.30 ± 28.16	< 0.001
**% of VASI change by anatomy localization**				
Face/Neck	65.06 ± 32.03	77.50 ± 32.48	52.02 ± 26.09	< 0.001
Trunk	70.00 ± 23.24	82.50 ± 8.22	55.00 ± 27.39	0.044
Limbs	62.27 ± 29.61	77.50 ± 16.96	44.00 ± 32.67	0.083
Acral	23.33 ± 37.83	37.00 ± 47.12	6.25 ± 12.50	0.224

*PGA* Physician Global Assessment, *VASI* Vitiligo area scoring index.

Trunk indicates: Chest, Abdomen, Back, Axilla.

### Patient and operator experience

**Figure 2:**
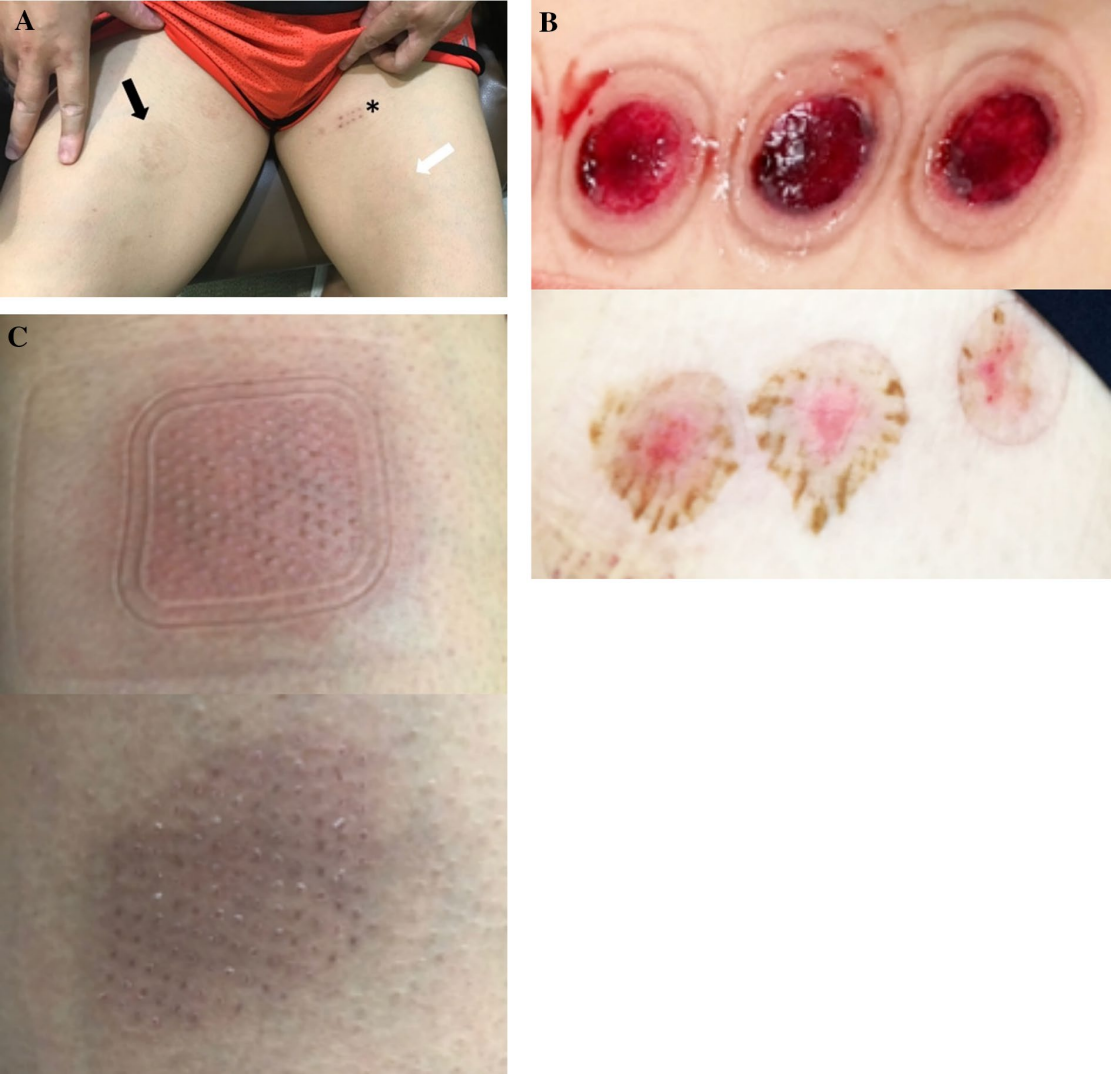
The donor site of a patient received all three procedures: SBEG, ABEM, and punch graft. (**A**) SBEG donor sites showed prolonged hyperpigmentation (12 months postoperatively, black arrow) in comparison to ABEM donor sites (third months postoperatively, white arrow) and scarring in punch graft (18 months postoperatively, asterisk). (**B**) (i) Immediate wound at the donor site of SBEG, a hematoma is sometimes noticed (ii) One week after surgery, some shallow abrasions are still visible. (**C**) (i) Immediate wound at the donor site of ABEM (ii) One week after surgery, wound healed entirely.

ABEM took significantly less time to harvest the blisters (SBEG: 74.74 ± 23.93 min, ABEM: 48.39 ± 21.07 min, p < 0.001) and to complete the whole procedure (SBEG: 112.87 ± 29.89 min, ABEM: 75.9 ± 27.93 min, p < 0.001) than SBEG (Table 3). The Visual Analogue Scale 10-point score for pain directly after the operation of ABEM was significantly lower than SBEG (SBEG: 5.39 ± 1.32, ABEM: 4.75 ± 1.10, p = 0.004). The duration of hyperpigmentation at the donor site was significantly longer in the SBEG group when comparing with the ABEM group (SBEG: 13.71 ± 5.51 months, ABEM: 3.54 ± 2.17 months, p < 0.001) (Fig. 2). Scar formation occurred in two participants who underwent SBEG, and no scarring event was found in patients treated with ABEM. No side effects such as cobble-stoning and the Koebner phenomenon were found. Interestingly, although the degree of repigmentation was better with SEBG, the improvement of DLQI was more profound in the ABEM group (p = 0.002).

**Table 3:** Patient and operator experience of conventional suction blister epidermal graft(SBEG) with automated blister epidermal micrograft (ABEM).

	Total	SBEG	ABEM	p-value
No. of anatomical based lesions	118	62	56	
Harvest time (minutes)	62.24 ± 26.11	74.74 ± 23.93	48.39 ± 21.07	< 0.001
Total procedure (minutes)	95.33 ± 34.09	112.87 ± 29.89	75.90 ± 27.39	< 0.001
**Donor site**				
Pain score (0–10)	5.08 ± 1.26	5.39 ± 1.32	4.75 ± 1.10	0.004
Hyperpigmentation (months)	8.80 ± 6.63	13.71 ± 5.51	3.54 ± 2.17	< 0.001
Scarring	2.00 ± 0.02	2.00 ± 0.03	0.00 ± 0.00	0.521
Post-operative changes in DLQI	3.98 ± 3.09	3.38 ± 3.33	4.64 ± 2.69	0.002

*Adjust age and gender.

### Patients received concomitant SBEG and ABEM

Thirty-eight anatomical-based lesions from ten patients underwent SBEG and ABEM treatments concomitantly, with 13 lesions receiving SBEG and 25 receiving ABEM (Table 4). The rate of repigmentation according to the PGA scale (p = 0.293) and the difference in VASI from baseline (p = 0.342) were not significant between the two groups. SBEG took a longer time to complete the entire process than ABEM (p = 0.051). For patient experience and donor side effects, the patient reported a higher pain score with SBEG (p < 0.001) and had prolonged hyperpigmentation at the donor site compare to ABEM (SBEG: 13.65 ± 5.28 months, ABEM: 3.40 ± 0.91 months, p < 0.001).

**Table 4:** The patient received concomitant conventional suction blister epidermal graft(SBEG) with automated blister epidermal micrograft (ABEM), n = 38.

	Total	SBEG	ABEM	p-value
No. of anatomical based lesions	38	13	25	
**Repigmentation PGA, n (%)**				0.293
Very good (≥ 75%)	20 (53%)	9 (69%)	11 (44%)	
Good (50–74%)	5 (13%)	1 (77%)	4 (16%)	
Fair (25–49%)	4 (11%)	0 (0%)	4 (16%)	
Poor (0–24%)	9 (24%)	3 (23%)	6 (24%)	
% of VASI change	95.13 ± 9.48	93.08 ± 13.77	96.20 ± 6.34	0.342
Total procedure (minutes)	92.26 ± 32.71	108.00 ± 36.32	84.08 ± 28.04	0.051
**Donor site**				
Pain score (0–10)	5.34 ± 1.24	6.38 ± 1.33	4.80 ± 0.76	< 0.001
Hyperpigmentation (months)	6.91 ± 5.82	13.65 ± 5.28	3.40 ± 0.91	< 0.001

*VASI* Vitiligo area scoring index.

## Discussion

Vitiligo is a common autoimmune skin disorder in which T-cells destroy melanocytes, resulting in depigmentation. It can lead to many social stigmas, which have a negative impact on one's mental health, including low self-esteem, a negative body image, and a substantial psychosocial burden^4,6^. Surgical therapies benefit stable vitiligo that failed to respond to medical treatment. SBEG is one of the most accessible and reliable surgical interventions available^19^. ABEM, a commercialized blister epidermal micrograft that automatically applies both heat and suction to the donor site, has been shown to reduce procedure time, minimize discomfort, and enhance the quality of life in a preliminary study^15,20^. Understanding the distinctions between various forms of vitiligo surgery and the benefits and drawbacks of each procedure are crucial for optimal surgical results.

We report a comparative study for refractory stable vitiligo treated with conventional SBEG versus ABEM. In our study, 83.87% of patients receiving SBEG achieved good to very good results (≥ 50% repigmentation), and 75.81% achieved very good results (≥ 75% repigmentation), which is comparable to the degree of repigmentation in a meta-analysis by Ju et al.^19^ In this study, SBEG was shown to be more effective than ABEM in terms of the degree of repigmentation as measured by PGA scale repigmentation rates and the difference in VASI from baseline. Based on our experience, this is caused by difficulties in fixation of the silicone-coated nonadherent dressing to the recipient site, particularly around lips, eyelids, and bony prominences. A subgroup analysis based on body parts supported this concept, revealing that the alterations in VASI were more pronounced in the SBEG group on the face/neck region than in the ABEM group. We also noticed that acral areas in both groups had the least amount of pigmentation spread, consistent with previous studies^21^. An optimal outcome may be achieved by developing or finding a more flexible and adhesive dressing that enhances proper fixation.

In terms of operator experience, ABEM took significantly less time to harvest the blisters and complete the procedure than SBEG, making it more convenient and appropriate for ambulatory surgery. It allows reduced operation time, which is beneficial for both operators and patients, especially children who cannot withstand long harvesting. SBEG, on the other hand, takes longer and is less suitable for broad achromic surfaces. The rounded blisters collected from SBEG must be tailored to the vitiligo area, which is sometimes uneven in shape and can result in graft waste. Furthermore, the quality of harvested blister is occasionally unpredictable, and the operator needs to pay close attention to the orientation of blisters to avoid engraftment problems caused by upside-down grafting. The ABEM contains micrografts aligned on silicone dressing that can be readily tailored to match the shape of the transplanted area and generate 20–25 cm^2^ of area in a single harvesting. Hence, the treatment area for each grafting with ABEM is larger than of SBEG.

**Figure 3:**
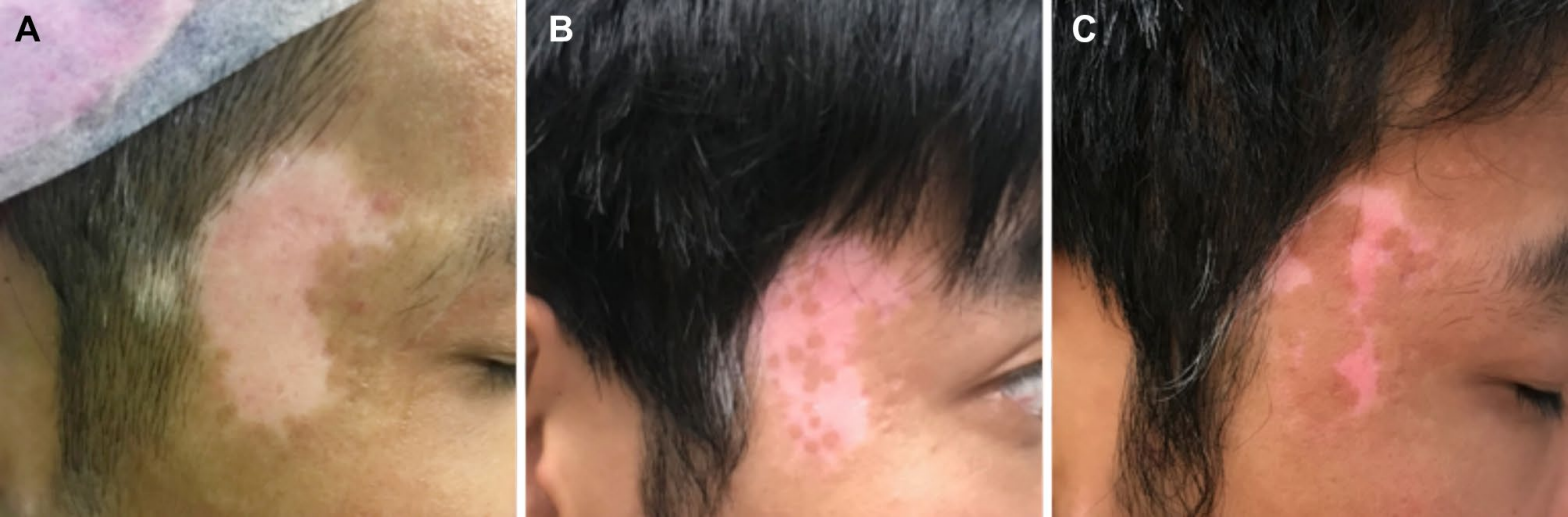
(**A**) Before surgery: A 5 × 4 cm^2^ depigmented patch in a 26 years-old stable, nonsegmental vitiligo patient who failed to respond to medical and excimer laser therapy. (**B**) One month after ABEM, dotted repigmentation corresponds to the micro-blister graft. (**C**) Three months after ABEM, the dotted repigmentation confluent into a patch with constant improvement over time.

In addition, when compared to SBEG, ABEM had better donor side outcomes, with patients experiencing significantly fewer side effects such as pain, donor site hyperpigmentation, and scarring. In ABEM, dotted repigmentation from the micrografts can be observed within one month and gradually expand centrifugally. Over time, the repigmentation confluent into a more homogenous pattern to cover the recipient area, with an optimal result at around a year after grafting (Fig. 3). On the contrary, the SBEG grafts are designed to cover the recipient area completely; hence, the repigmentation requires less time to cover the entire recipient area completely.

**Figure 4:**
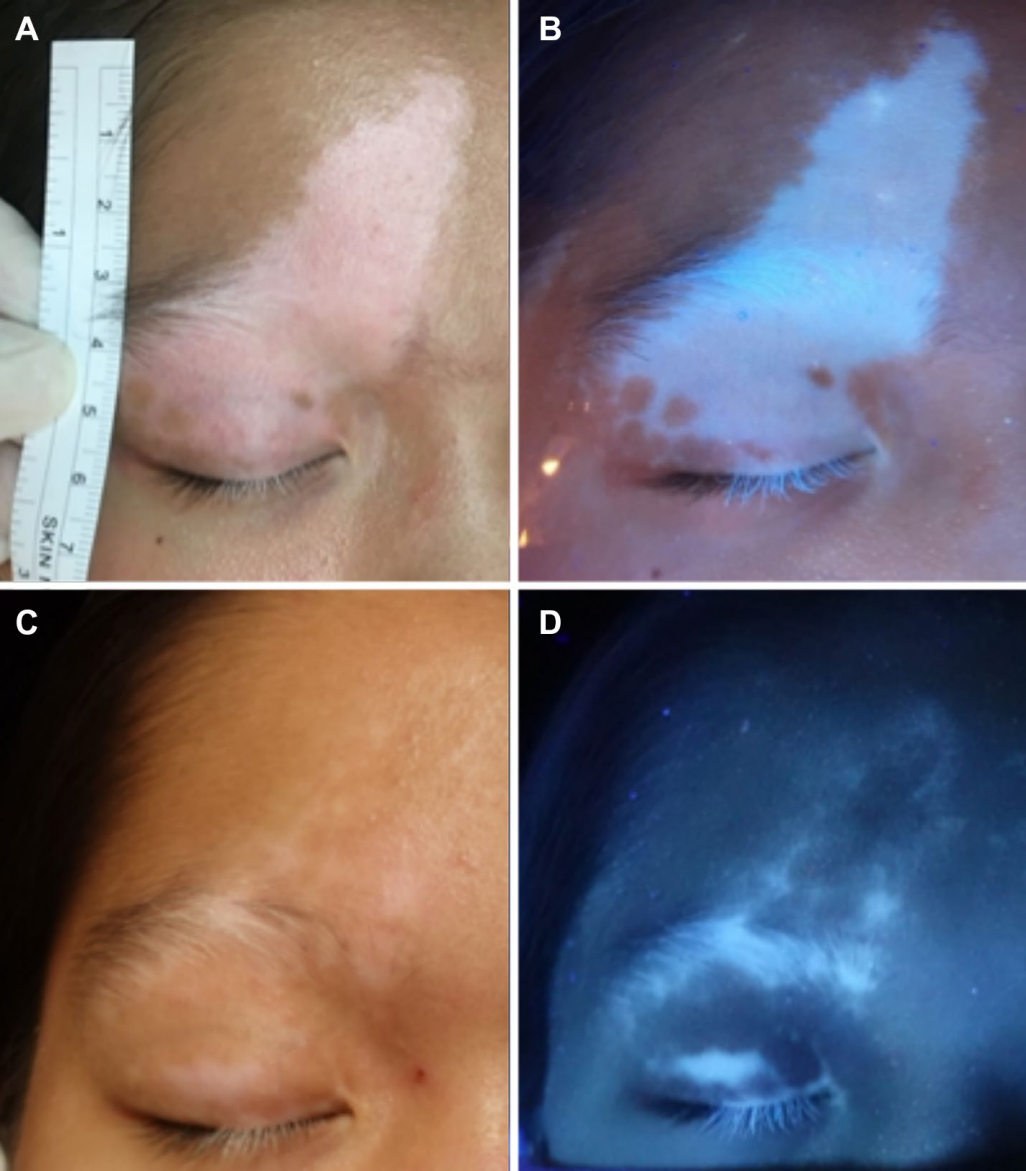
A 10 years-old girl with segmental vitiligo received combination procedures: ABEM on the upper forehead and SBEG for the upper eyelids. (**A**-Room light; **B**-Woods' lamp; **C**-Room light, one year after surgical grafting; **D**-Wood’s lamp, one year after surgical grafting.)

Surprisingly, despite the fact that SBEG had a superior degree of repigmentation and is less expensive, the ABEM reported a more significant improvement in life quality. This result may be due to improved operational experience and fewer negative consequences on the donor side. When we looked at patients who had undergone both procedures, we discovered that ABEM took less time and had fewer side effects than SBEG. Furthermore, our beliefs often affect how we experience medical treatment. The subject-expectancy effect of a better outcome due to ABEM's higher price may explain this discrepancy^22,23^. ABEM has a higher yield of graft area (20–25 cm^2^) per harvest than SBEG, making it suitable for a larger recipient area. In the intra-individual study, there was little difference in repigmentation efficacy between SBEG and ABEM. Therefore, ABEM can be an alternative for individuals seeking a less painful and more convenient treatment modality. Furthermore, combining the two treatments to optimize the surgical outcome may be an option (Fig. 4).

There are some limitations to this study. The results are limited to a single operator in a tertiary medical center. Besides, this is a retrospective comparative study. Future randomized and split-control investigations are needed to confirm the findings of this study.

## Conclusion

To the best of our knowledge, this is the first study directly comparing the effect and safety for SBEG and ABEM. We found that stable vitiligo treated with SBEG had a significantly higher repigmentation rate than those treated with ABEM, particularly around lips, eyelids, and bony prominences. SBEG should be used on the face and neck because of its better efficacy and cost-effectiveness. ABEM, on the contrary, has some advantages, including better blister quality, a higher yield of graft area (20–25 cm^2^) at a single harvest, and a better operational experience, which includes a shorter procedure time, fewer donor side problems (scarring, prolonged hyperpigmentation), and improved quality of life. Understanding the benefits and drawbacks of two blister grafting methods is crucial for optimal surgical results.
